# Association of Exosomal miR-210 with Signaling Pathways Implicated in Lung Cancer

**DOI:** 10.3390/genes12081248

**Published:** 2021-08-16

**Authors:** Qiaoyi Chen, Xiaoge Xie

**Affiliations:** Department of Cell Biology and Genetics, School of Basic Medical Sciences, Xi’an Jiaotong University, Xi’an 710049, China; xiexiaoge@snnu.edu.cn

**Keywords:** micro-RNA, miR-210, exosomes, lung cancer

## Abstract

MicroRNA is a class of non-coding RNA involved in post-transcriptional gene regulation. Aberrant expression of miRNAs is well-documented in molecular cancer biology. Extensive research has shown that miR-210 is implicated in the progression of multiple cancers including that of the lung, bladder, colon, and renal cell carcinoma. In recent years, exosomes have been evidenced to facilitate cell–cell communication and signaling through packaging and transporting active biomolecules such as miRNAs and thereby modify the cellular microenvironment favorable for lung cancers. MiRNAs encapsulated inside the lipid bilayer of exosomes are stabilized and transmitted to target cells to exert alterations in the epigenetic landscape. The currently available literature indicates that exosomal miR-210 is involved in the regulation of various lung cancer-related signaling molecules and pathways, including STAT3, TIMP-1, KRAS/BACH2/GATA-3/RIP3, and PI3K/AKT. Here, we highlight major findings and progress on the roles of exosomal miR-210 in lung cancer.

## 1. Introduction

Over the past few decades, tremendous strides have been made in understanding the genetics and treatment of lung cancer. However, lung cancer remains the prevailing cause for global cancer-related morbidity and mortality [1]. Lung cancer is classified into two histological subtypes: non-small cell lung cancer (NSCLC) and small cell lung cancer (SCLC) [2]. NSCLC, which includes adenocarcinoma, squamous cell carcinoma, and large cell carcinoma, is the most prevalent, covering approximately 80% of all lung cancer cases [3]. SCLC is less commonly found (15–20%) but is known to proliferate and metastasize more rapidly than NSCLC. In addition to these two main types, rare lung tumors such as carcinoid tumors, adenoid cystic carcinomas, sarcomas, and benign hamartomas have also been reported. Despite a wide array of currently available treatment methods including surgery, radiotherapy, chemotherapy, and immunotherapy, the 5-year survival rate of lung cancer patients is still under 20% [4]. Poor disease prognosis is in part due to limited understanding of the complex nature of lung tumor heterogeneity as well as late disease presentation and diagnosis. Notably, cancers are known to have long incubation periods (~20 years), during which time, the sensitivity of typical detection methods such as ultrasound, x-ray-based computer tomography, and endoscopy are inept. In recent years, liquid biopsy has become a widely used technique in clinical settings due to its ease of use, minimal invasiveness, and low cost. Most important, genomic information, such as global gene expression dysregulation, extracted from biofluids provide higher accuracy for disease detection as well as insights for underlying mechanisms of disease pathogenesis.

Aberrant expression of microRNAs (miRNAs) has been well-documented in lung cancer. Elevated oncogenic or reduced tumor suppressive miRNAs are equally important in altering cancer-related signaling pathways, and have been implicated in tumor cell growth, angiogenesis, and metastasis. In body fluids, miRNAs exist as circulating Ago protein-bound forms that are either released from damaged and dead cells or selectively packaged into extracellular vesicles (EVs) for cell signaling purposes [5,6,7]. Exosomes have been evidenced to play an important role in mediating cell–cell communication through transferring and depositing active biomolecules such as miRNAs, thereby eliciting epigenetic changes in recipient cells. Various exosomal miRNAs are dysregulated in lung cancer. In particular, miR-21, miR-31, and miR-192 are most commonly found in human lung cancer tissues and blood samples [8,9,10,11]. Through a comprehensive literature search, we find that aberrant expression of exosomal miR-210 is found across various human, cell, and animal models of lung cancer, indicating an important role in cancer development. MiR-210 is a peculiar miRNA; apart from various cancers, its dysregulation is also associated with other human diseases such as cardiovascular disease and diabetic obesity [12,13]. What is more interesting is that its inclusion in exosomes in response to hypoxia is also relevant in placental disorder preeclampsia [14]. This review aims to examine the role of exosomal miR-210 in lung cancer and its potential underlying pathways. 

## 2. Biogenesis and Function of miRNAs 

MiRNAs are a group of endogenous non-coding RNAs approximately 22 nucleotides in length, and mainly function to mediate post-transcriptional gene silencing by binding to complementary sites in the 3′ untranslated region (UTR) of target mRNAs. Processing of miRNA can happen post- or co-transcriptionally [15,16]. Intragenic miRNAs are generated mostly from introns of protein coding genes, while intergenic miRNAs, located between genes, are processed by their own RNA polymerase II or III promoters [17]. MiRNAs are known to have short seed regions, approximately seven nucleotides, that are often found to be similar to a multitude of other miRNAs. In general, miRNA can be processed through either canonical or non-canonical pathways [16,18,19]. Non-canonical miRNA biogenesis includes Dicer-independent and Drosha/DGCR8-independent pathways. Pre-miRNAs produced such as mirtrons often resemble Dicer substrates. In the canonical pathway, the dominant pathway, pri-miRNAs transcribed by RNA polymerase II are first cut into pre-miRNAs by microprocessor complex, which consists of RNA binding protein DiGeorge Syndrome Critical Region 8 (DGCR8) that recognizes pri-miRNA motifs, and Drosha, a ribonuclease III enzyme responsible for cleaving the pri-miRNA duplex, leaving a 3′ overhang [20,21]. Lastly, pre-miRNA is transported to the cytoplasm via exportin 5 (XPO5)/RanGTP complex, where the terminal loop is removed by RNase III endonuclease Dicer, resulting in mature miRNA duplex [22]. The double helix is then unwound by helicase into either 3p or 5p strands. The 3p strand originates from 3′ end of the pre-miRNA hairpin, and 5p strand comes from the 5′ end. While both strands can be loaded into Argonaute (AGO) and serve as the guide strand, the preferred strand is often the one with the lower 5′ thermodynamic stability, mostly with uracil at the 5′ end. The leftover or passenger strand will then be eliminated by AGO-related degradation mechanisms. 

MiRNAs exist in the genome as single copies, multiple copies or gene clusters, have fixed gene loci, and are highly conserved through evolution. In animals, miRNA seed regions, which span 2–8 nucleotides at the 5′ end, are critical for mRNA recognition. Considering the short seed region, individual miRNAs can target hundreds or even thousands of different mRNAs, and similarly, individual mRNAs can be coordinately suppressed by various miRNAs. However, despite seemingly non-selective binding activity of miRNAs, reports that certain genes are preferentially targeted suggest that miRNA-led inhibition may not be of random chance, but through an unknown yet sophisticated targeting mechanism. While in silico analyses provide predicted targets, the standard confirmation for miRNA–mRNA binding is through biological assay, where a luciferase reporter fused to 3′UTR is reduced by miRNA overexpression or expressed when there is a point mutation in the 3′UTR. Unlike animals, in most plants, miRNA is perfectly complementary to the 3′ UTR or even the coding region of the target mRNA, and can cleave target mRNA in the complementary region, leading to gene silencing. MiRNAs are also known to be tissue-specific. For example, in Arabidopsis thaliana, miR-157 is highly expressed in seedlings and miR-171 is highly expressed in flowers. Interestingly, the first miRNA, Lin-4, was discovered by Ambros and Ruvkun in *Caenorhabditis elegans* in 1993, but it was not until the early 2000s that miRNAs were formally classified as a distinct group of non-coding biological regulators [23,24,25,26]. Since then, the field of miRNA has taken off, spanning a wide range of study areas including molecular cancer biology, immunology, neurology, etc. Today, it is well known that normal function of miRNAs in the human body is necessary for maintenance of cellular homeostasis [27,28]. MiRNAs can influence the occurrence, development, and formation of diverse diseases by regulating cell proliferation, apoptosis, cell differentiation, cell cycle, hormone secretion, and biological development [27,29,30]. MiRNAs have been evidenced to participate in a multitude of cellular activities, including cell fate determination, proliferation, apoptosis, immune response circadian rhythm, viral replication, hormone secretion, etc. [24,31,32,33,34,35]. For example, miR-29a plays a crucial role in the dysregulation of metabolism and inflammatory signaling linked to NAFLD severity and progression [36]. The miR-15 family is capable of promoting cell cycle arrest and suppressing mitotic genes [37]. MiR-155 upregulation represents an important signal in various inflammatory diseases, some clinical trials have suggested this association as a biomarker for inflammation [38]. Downregulation of miR-146a may contribute to severe COVID-19 symptoms in patients suffering from diabetes, obesity, and hypertension [39]. MiRNAs have also been proposed to serve as early diagnostic biomarkers for identifying high-risk subjects and early cancer stages [40]. Large-scale miRNA dysregulation can be found in almost all cancer types. In particular, accumulating evidence has revealed that exosomal miRNA plays a critical role in mediating favorable microenvironment for lung cancer development. More importantly, tumor cells have been evidenced to promote an immunosuppressive and chemoresistant environment through exosomal regulation, part of which may be due to the role of miRNA in actively regulating the epigenetic landscape. 

## 3. Exosomes

Exosomes are extracellular vesicles released by living cells, with a diameter ranging between 30~100 nm, and a density between 1.13–1.19 g/mL [41,42,43]. Exosomes were initially described by Johnstone as being uniquely secreted by reticulocytes [44,45]. The main biogenesis mechanism is through the invagination of intracellular lysosomal particles, which is released into the extracellular space after fusing with the cell membrane [46,47,48,49,50]. It is found that almost all cells can secrete exosomes under both normal and pathological conditions, including reticulocytes, dendritic cells, lymphocytes, platelets, mast cells, and tumor cells [44,51]. Exosomes can be detected in urine, cerebrospinal fluid, saliva, sputum, serum, plasma, milk, semen, pleural effusion, amniotic fluid, and other biological fluids [46,52,53,54,55,56,57]. While the composition of exosomes is similar to and determined by their parental cells, it is interesting to note that exosomes released by all cell types contain several common marker proteins, including CD63, CD81, CD9, TSG-101, and ALIX [47,58,59]. 

Exosomes are efficient in transmiting signals and transferring biologically active molecules such as proteins, growth factors, cytokines, RNAs, DNAs, and lipids [46,48,51,60,61]. During the transferring process, these biologically active molecules are surrounded by a lipid bilayer membrane composed of cholesterol, phosphatidylserine, and sphingolipids, and thus protected from degradataion. Active contents propageted by exosomes can serve as stable signaling molecules mediating cell–cell communication and participating in various cancer-related biological activities including cell migration, angiogenesis, pre-metastasis niche formation, drug resistance, and immune regulation [41,62,63,64,65,66,67,68,69,70]. The secretion and transportation of exosomes are accomplished through two main pathways. The first mechanism requires the endosomal sorting and transport complex (ESCRT). ESCRT is composed of four proteins (ESCRT-0, ESCRT-I, ESCRT-II, and ESCRT-III), and functions to package biomolecules in the internal cavity of exosomes. The other secretion method is the ESCRT-independent mechanism, which requires the help of sphingolipids [41,44,71,72]. Once released by the parent cell, exosomes can interact with neighboring or distant cells through at least three mechanisms: (i) interaction between the exosomal transmembrane protein and the signal receptor of the recipient cell, (ii) fusion with the plasma membrane of the recipient cell and release of their contents into the cytosol, (iii) internalization of exosomes into the recipient cell through endocytosis [73]. Studies have shown that exosomal miRNAs released in the cytoplasm are functional and may induce changes in the function and phenotype of recipient cells [74,75]. Existing data show that packaging of microribonucleic acid into exosomes is a selective process. When tumor cells develop, levels of specific exosomal microribonucleic acids will change. For these reasons, exosomal miRNA can also act as potential candidate biomarkers for clinical applications [76]. Accumulating evidence shows that exosomal miRNAs, especially miR-210, may play a key role in changing the microenvironment of lung cancer, and may promote progression, invasion, angiogenesis, metastasis, and drug resistance. 

## 4. MiR-210 Function in Cancer

MiR-210 exists in two forms: miR-210-3p (guide strand) and miR-210-5p (passenger strand). Most studies in literature focus on miR-210-3p since it can mature into functional miRNA and integrate with the RNA-induced silencing complex (RISC), while the passenger strand is meant for degradation [77,78]. MiR-210 is highly involved in a variety of biological processes including mitochondrial metabolism, angiogenesis, cell proliferation, apoptosis, and erythropoiesis [79,80,81,82]. MiR-210-3p has shown both oncogenic and tumor suppressive properties. MiR-210 expression is elevated in renal cell carcinoma (RCC), hepatocellular carcinoma, breast cancer, colorectal cancer, pancreatic cancer, and lung cancer [83,84]. In RCC patients, miR-210-3p levels are found to be especially high in urine [85]. In bladder cancer, miR-210-3p has been evidenced to inhibit tumor growth by targeting fibroblast growth factor receptor-like 1 (FGFRL1) and promote prostate cancer cell epithelial–mesenchymal transition (EMT) and bone metastasis by targeting the NF-kB signaling pathway [86]. In colon cancer patients, high miR-210 levels are correlated with metastasis and poor prognosis [87]. Moreover, in colorectal and hepatocellular carcinoma cells, overexpression of miR-210 has been evidenced to inhibit vacuole membrane protein 1 (VMP1) and enhance migration and invasion [88,89,90,91]. 

Target prediction software such as TargetScan and PicTar have revealed various potential miR-210 targets, including E2F transcription factor 3 (E2F3), RAD52, and Max’s Next Tango (MNT), and reflect the potential roles of miR-210 in regulating cell cycle, proliferation, and genome integrity [78]. MNT is a transcription factor that competes with c-MYC for max binding and is critical for cell cycle progression [92,93,94]. miR-210 has been evidenced to bind to the 3′UTR region of MNT under hypoxic conditions to inhibit its transcription and indirectly promote c-MYC activation and cell cycle progression [79]. Conversely, knockdown of miR-210 can lead to the overexpression of MNT and cell cycle arrest, as shown in glioma stem cells [95]. Furthermore, miR-210 has been demonstrated to inhibit E2F3 expression, which is known to preferentially target tumor suppressor genes and inhibit cell proliferation [96,97]. In addition to cell cycle control, miR-210 has also been implicated in disruptions of DNA damage repair pathways. For example, cells overexpressing miR-210 exhibit low levels of RAD52 and greater instances of DNA strand breaks [98]. Similar to BRCA2, RAD52 serves to recruit RAD51 to repair double-strand breaks through nucleoprotein filament formation [89,97,99,100]. 

In vitro studies have found that miR-210-induced VEGF expression promoted cancer cell migration and angiogenesis [101]. Angiogenesis, a process of new blood vessel formation, is particularly important for cancer cell proliferation and metastasis. Hypoxia-induced miR-210 overexpression triggers lactic acid fermentation and increase in glucose transporter GLUT-1. GLUT-1 upregulation is often found coupled with vascular endothelial growth factor (VEGF) and platelet-derived growth factor (PDGF) expression, creating favorable conditions for angiogenesis [102]. Alternatively, miR-210 can also promote VEGF expression through the inhibition of phosphor-tyrosine phosphatase-1B (PTP1B) and Ephrin-A3(EFNA3) [98]. Moreover, its role in regulating hypoxia-inducible factors (HIFs) under low oxygen (hypoxic) conditions is well documented. Notably, hypoxia is a common feature found in many cancer types, as the demand for oxygen in proliferating tumor tissues eventually exceeds the supply. Under normal circumstances, glycerol-3-phosphate dehydrogenase 1-like (GPD1L) activates prolyl-hydroxylase domain isoforms (PHDs) to promote HIF-1 proline hydroxylation and proteasomal degradation. However, under hypoxic conditions, elevated miR-210 expression inhibits GPD1L through mRNA 3′UTR binding, and results in HIF-1 accumulation [103,104]. HIF-1 accumulation can up-regulate CA9, COX2, and VEGFA, thereby adapting to hypoxic conditions [105]. Noted, miR-210-HIF-1 interaction is bi-directional. When the oxygen level decreases, increase in HIF-1 protein can also promote miR-210 accumulation, indicative of a positive feedback loop. Moreover, upon hypoxic stress, miR-210 can inhibit DNA repair factor RAD52, increase ROS production, and decrease mitochondrial respiratory activity [77,79,106]. Notably, hypoxia can also promote exosomal miR-210 release through increased production of tissue inhibitor of metalloproteinases-1 (TIMP-1). TIMP-1 has been evidenced to promote angiogenic tubulogenesis through upregulation of miR-210 expression via the PI3K/AKT pathway [107]. Current studies indicate that exosomes excreted by hypoxic cancer cells may be responsible for promoting tumor progression via miR-210 secretion [108,109]. For example, circulating exosomes isolated from breast cancer and colorectal cancer patients show increased miR-210 expression [90,110]. 

## 5. Mechanisms of Exosomal miR-210 in Lung Cancer

In 2009, Rabinowits et al. first reported that miRNAs extracted from NSCLC tissue can serve as diagnostic biomarkers [111,112]. Since then, various miRNAs have been implicated in the development of lung cancer, and miR-21 has been one of the most extensively studied candidates. However, while dysregulation of exosomal miR-210 has been reported in human, cell, and animal studies (Table 1), less is known about its underlying mechanisms in lung cancer. This section will examine all currently known mechanistic pathways involved in exosomal miR-210-mediated lung cancer.

### 5.1. Signal Transducer and Activator of Transcription 3 (STAT3)

Hypoxic bone marrow-derived mesenchymal stem cells (BMSCs) have been evidenced to transfer exosomal miRNAs to promote lung cancer metastasis. Specifically, lung cancer cells (A549, LLC, H460, and H358) treated with hypoxic BMSC-derived exosomes demonstrated increased migration and invasion potentials compared to normoxic BMSC-secreted exosomes [114]. Hypoxic BMSC-derived exosomes were especially rich in miR-193a-3p, miR-210-3p, and miR-5100. Furthermore, BMSC-derived exosomes promoted both the total and phosphorylated STAT3 levels [114]. STAT3 is known to be overexpressed in cancer cells, and functions to elicit production of immunosuppressive factors. Moreover, miR-210-3p inhibitor was capable of reducing phosphorylated STAT3 expression. The study further analyzed plasma exosomes and found significantly upregulated miR-210-3p levels in metastatic lung cancer patients compared to non-metastatic lung cancer patients and healthy controls, suggesting that miR-210 may play an important role in lung metastasis. Specifically, miR-210-3p is capable of targeting STAT3 inhibitor, suppressor of cytokine signaling 1 (SOCS1) [115]. Interestingly, miR-210-5p has also been shown to directly target SOCS1 in RCC [116]. 

### 5.2. Fibroblast Growth Factor Receptor Like 1 (FGFRL1)

Cancer cells have high heterogeneity and contain a variety of cell types. Cancer stem cells (CSCs) for example, make up a small population of cancer cells, and are characterized by enhanced self-renewal and chemo/radiotherapy resistance capabilities, which make them the main mediators for sustained cancer growth. Lung CSC-derived exosomes have been evidenced to contain high levels of miR-210-3p and enhance lung cancer cell migration and invasion, through the inhibition of E-cadherin as well as the promotion of vimentin, N-cadherin, MMP-9, and MMP-1 expression, which are phenotypic hallmarks for EMT and enhanced invasive potential [103]. Moreover, the study indicated that miR-210-3p may contribute to cancer cell metastasis via the inhibition of FGFRL1. FGFRL1 is part of the FGFR family and has been reported to modulate ERK1/2 and FGF signaling pathways [117]. Recently, FGFRL1 has been associated with prostate, gastric, oesophageal, and ovarian cancer cell proliferation and metastasis [118,119]. In particular, miR-210 has been evidenced to promote angiogenesis by targeting FGFRL1 in hepatocellular carcinoma and osteosarcoma cells [120,121]. However, in oesophageal squamous cell carcinoma, laryngocarcinoma, and bladder cancer, miR-210-3p has showed tumor suppressive properties through FGFRL1 binding [91,120,122]. These conflicting results suggest that miR-210-3p and FGFRL1 may have dual roles in cancer.

### 5.3. PI3K/AKT Pathway

Runt-related transcription factor-3 (RUNX3) is primarily involved in cartilage mineralization and chondrocyte maturation, though evidence suggests that miRNA-regulated RUNX3 is capable of influencing phosphatidylinositol-3-kinase protein kinase B (PI3K/AKT) signaling pathway, which is crucial for cancer cell proliferation [123,124,125]. RUNX3 is correlated with poor prognosis and shorter survival in NSCLC patients [126,127]. A study led by Li et al. reported that miR-210 was capable of inhibiting RUNX3, thereby activating PI3K/AKT signaling pathway and promoting malignant phenotype of lung cancer cells [127]. Conversely, the inhibition of miR-210 or PI3K/AKT signaling pathway via LY294002 treatment reversed malignant potential of lung cancer cells. In addition to RUNX3, PTEN is another well-known regulator of the PI3K/AKT signaling pathway. For example, overexpression of miR-210 has been shown to promote NSCLC cell migration and invasion through UPF1 suppression followed by upregulation of the PTEN/PI3K/AKT pathway [128]. More recently, miR-210 upregulation has been reported to inhibit upstream stimulating factor 1 (USF-1) and polycomb group ring (PCGF3) [129]. USF-1 is a transcription factor belonging to the basic helix-loop-helix leucine zipper family, and is known to regulate hepatocellular carcinoma, papillary thyroid as well as lung cancer [130]. Interestingly, PCGF3 has also been reported to promote cell proliferation in NSCLC via the PI3K/AKT signaling pathway [131]. Moreover, miR-210-mediated PI3K/AKT signaling has also been reported in oral cancer. Notably, in oral squamous cell carcinoma, elevated exosomal miR-210-3p levels can inhibit ephrinA3 expression and in turn activate PI3K/AKT signaling pathway [132]. Overall, these studies suggest that miR-210 can alter PI3K/AKT through various factors, and that this phenomenon is not limited to lung cancer.

### 5.4. Tissue Inhibitor of Metalloproteinases-1 (TIMP-1)

TIMP-1 is known to regulate protease homeostasis via the inhibition of metzincin [133,134]. Its ability to inhibit matrix metalloproteinases (MMPs) and A-disintegrin-and-metalloproteinase (ADAM-10) reflect anti-tumorigenic characteristics. However, increased TIMP-1 expression is often correlated with poor prognosis, especially in ovarian, lung, gastric, and papillary thyroid carcinoma [135,136,137,138,139,140]. Interestingly, TIMP-1 serves as a positive regulator of PI3Ks and has been evidenced to promote cancer cell growth via AKT/ERK phosphorylation [141,142,143,144,145]. A study led by Cui et al. showed that an increase in TIMP-1 promoted lung cancer progression through activating the PI3K/AKT/HIF-1 signaling pathway and miR-210 expression [107]. Specifically, high levels of miR-210 were found in exosomes derived from TIMP-1 overexpressing A549L cells, and that its expression level was dependent on HIF-1 accumulation. Conversely, a reduction in miR-210 can effectively inhibit A549L cell growth, suggesting its important role in cancer cell proliferation. Previous research has reported that hypoxia promotes exosome secretion of miR-210, suggesting a mechanism of a self-sustaining hypoxia state. Moreover, the study finds that levels of mature miR-210 was dependent on CD63, an interacting partner of TIMP-1, providing novel insight into the mechanism of elevated miR-210 in lung cancer. 

### 5.5. Epidermal Growth Factor Receptor (EGFR)-Mutant Drug Resistance

Osimertinib is a tyrosine kinase inhibitor, specifically designed to treat EGFR-mutant non-small cell lung cancer [146,147]. Despite its effectiveness compared to previous two generations of EGFR-tyrosine kinase inhibitors (EGFR-TKIs), multiple studies have reported resistance to osimertinib, due to varying mechanisms, including EGFR mutation, KRAS mutation, BRAF mutation, loss of T900M mutation, or HER2 amplification [148]. Using microarray and qRT-PCR, Hisakane et al. reported high levels of exosomal miR-210 in osimertinib-resistant HCC827-OR and PC-9-OR cells compared to HCC827 and PC-9 parental cells [113]. Moreover, co-culturing exosomes isolated from osimertinib-resistant cells as well as induction of miR-210 both led to drug resistance and EMT in oximertinib-sensitive cells. However, there was no evidence that miR-210 acted via the EGFR signaling pathway, suggesting the involvement of a bypass mechanism. The study points to E-cadherin as a potential mediating factor associated with EMT. In addition, exosomes isolated from colorectal cancer cells and pancreatic cancer stem cells have also been found to carry high abundance of miR-210 and are correlated with fluorouacil and gemcitabine resistance [149,150,151]. 

### 5.6. KRAS BACH2/GATA-3/RIP-3

Mutant KRAS is a well-known driver of lung neoplasia, part of which functions through secreting exosomes to manipulate tumor microenvironment favorable for hypoxic immunosuppression [152,153,154]. Interestingly, in KRAS chemoresistant lung cancer tissues from human patients, high abundance of miR-146 and miR-210 were found compared to non-KRAS metastatic samples [154]. Moreover, post KRAS exosome inhibition, miR-210 expression levels were reduced, suggesting a direct relationship between KRAS and miR-210 levels. In addition, levels of miR-146/miR-210 were found at lower levels in lymph node metastatic tissues, indicating their importance in primary lung tumor. The study went on to report that KRAS was capable of regulating chromatin remodeling genes SMARCE1/NCOR1, which play key roles in chemosresistant metastasis, as well as transcription factor BACH2/GATA-3 expression through pyruvate/PKM2-dependent metabolism, thereby contributing to sustained immunosuppressive metastasis [154]. Although the mechanism of how miR-210 is regulated by KRAS remains elusive, there is clear evidence that PKM2 is an HIF-1 target gene [155]. 

## 6. Conclusions

Studies on exosomal miRNA represent a growing niche for cancer biology. While human and animal model studies use similar methods including miRNA-seq, qRT-PCR for the purpose of finding potential diagnostic biomarkers, there are only a few consistent results among currently available studies. The vast number of dysregulated miRNAs reported makes it difficult to pinpoint which miRNA is responsible for the development of lung cancer. Currently, there is a limited amount of research on the role of exosomal miR-210 in lung cancer, but dysregulation of its expression has been reported in various human, cell, and animal models. The consistency in its dysregulated expression found under various contexts suggests that miR-210 may play an important role in lung cancer development. MiR-210 is a well-known hypoxia-related miRNA and has been evidenced to mediate both oncogenic and tumor suppressive properties. Confirmed miR-210 down-stream targets include STAT3 and FGFRL1; however, less is known about its upstream targets. Future studies may benefit from investigating probable miR-210 regulators as well as potential exosomal inhibitors and anti-miR-210 agents for therapeutic purposes.

## Figures and Tables

**Table 1:** MiR-210 expression in human, cell, and mouse models.

Exosomal miRNA	miR-210	miR-210-3p	miR-210-3p	miR-210-3p	miR-210	miR-210
Expression Level	Up	Up	Up	Up	Up	Up
Sample Source	Human	Cell	Cell	Cell	Cell	Fox Chase SCID mice
Sample Type	Pleural effusion	HCC827 cells, PC-9 cells, HCC827-OR cells, PC-9-OR cells	H358 cells, A549 cells, H460 cells	A549 cells, NCIH1703 cells, BEAS-2B cells	A549 cells, HEK-293/EBNA cells	Plasma
Exosome Isolation Method	Exosome isolation reagents (Invitrogen)	differential centrifugation	EXO Quick	ultracentrifugation	ExoQuick-TC	ExoQuick-TC
miRNA Detection Method	qRT-PCR	miRNA microarray and qRT-PCR	miRNA microarray	qRT-PCR	qRT-PCR	qRT-PCR
Upstream Regulator	unknown	unknown	unknown	unknown	TIMP-1	TIMP-1
Downstream Target	unknown	unknown	STAT3 signalling	FGFRL1	EphA3	FGFRL1, E2F3, VMP-1, RAD52 and SDHD
Function	unknown	Drug resistance	Invasion, Metastasis, EMT	pro-proliferative	Angiogenesis	Vascularization
Cancer Type	adenocarcinoma	NSCLC	NSCLC	Not specified	adenocarcinoma	adenocarcinoma
Reference	[9]	[113]	[114]	[103]	[102]	[107]

## Data Availability

Not applicable.
